# Chronic migraine classification: current knowledge and future perspectives

**DOI:** 10.1007/s10194-011-0393-6

**Published:** 2011-10-26

**Authors:** Gian Camillo Manzoni, Vincenzo Bonavita, Gennaro Bussone, Pietro Cortelli, Maria Carola Narbone, Sabina Cevoli, Domenico D’Amico, Roberto De Simone, Paola Torelli, On behalf of ANIRCEF (Associazione Neurologica Italiana Ricerca Cefalee)

**Affiliations:** 1 Department of Neurosciences, Headache Centre, Istituto di Neurologia, University of Parma, c/o Azienda Ospedaliero-Universitaria (Padiglione Barbieri, 3° piano), Via Gramsci 14, 43100 Parma, Italy; 2 University “Federico II” of Naples, Naples, Italy; 3 Clinical Neurosciences Department, C. Besta National Neurological Institute, Milan, Italy; 4 Department of Neurological Sciences, University of Bologna, Bologna, Italy; 5 Department of Neurosciences, Psychiatric and Anaesthesiological Sciences, University of Messina, Messina, Italy

**Keywords:** Chronic migraine, Transformed migraine, Chronic daily headache, Chronic headache, Headache, Migraine

## Abstract

In the field of so-called chronic daily headache, it is not easy for migraine that worsens progressively until it becomes daily or almost daily to find a precise and universally recognized place within the current international headache classification systems. In line with the 2006 revision of the second edition of the International Classification of Headache Disorders (ICHD-2R), the current prevailing opinion is that this headache type should be named chronic migraine (CM) and be characterized by the presence of at least 15 days of headache per month for at least 3 consecutive months, with headache having the same clinical features of migraine without aura for at least 8 of those 15 days. Based on much evidence, though, a CM with the above characteristics appears to be a heterogeneous entity and the obvious risk is that its definition may be extended to include a variety of different clinical entities. A proposal is advanced to consider CM a subtype of migraine without aura that is characterized by a high frequency of attacks (10–20 days of headache per month for at least 3 months) and is distinct from transformed migraine (TM), which in turn should be included in the classification as a complication of migraine. Therefore, CM should be removed from its current coding position in the ICHD-2 and be replaced by TM, which has more restrictive diagnostic criteria (at least 20 days of headache per month for at least 1 year, with no more than 5 consecutive days free of symptoms; same clinical features of migraine without aura for at least 10 of those 20 days).

## Introduction

The 1980s were a seminal period for the study of primary headache, as they were characterized not only by a wealth of investigational activity and significant progress in the knowledge of the disease, but above all by an increasingly rigorous approach from the scientific point of view.

The decade opened with the results of the Danish studies [1, 2] on regional cerebral blood flow (rCBF) in migraine—which was still named “classic” back then and is now known as “with aura”. These studies paved the way for the trigeminovascular theory, which is still considered a good explanation for the underlying pathogenetic mechanisms of the disease. Then, at the close of the decade came a number of accurate controlled trials that demonstrated the efficacy of sumatriptan in the symptomatic treatment of migraine [3], leading to the introduction of a new class of drugs that are still considered the therapy of choice for migraine attacks. In between, there were two all-important steps: the joint commitment from researchers of several countries to draw up the first version of the International Headache Society (IHS) classification, which was published in 1988 [4] and represented a true cultural and practical turning point; and the first demonstrations of investigators’ interest for those migraine patients who over time experience a progressive increase in the frequency of attacks until they develop a so-called chronic daily headache (CDH) [5, 6].

Among headache experts, the debate on CDH has been heated and controversial since the very beginning and is not yet over. To sum up the question in a few words, we can say that the issue revolved around two antithetic views.

On the one hand, there were those who thought that, in the wide-ranging headache chapter, CDH was a group that naturally included different subtypes—some of which still undefined—but was nonetheless autonomous and therefore should be considered, assessed and classified independently of the other primary headache forms [6–8].

On the other hand, there were those who thought that CDH was nothing but an entirely generic definition, a hotchpotch containing a non-homogeneous group of disparate headache forms that had already found their place in the current classification systems: in their opinion, this was a non diagnosis, or a diagnosis still waiting to be defined, if not a diagnosis made by unskilled people [9, 10].

Both views were based on elements and remarks that were worthy of the utmost respect. The former view was more closely related to clinical practice, the latter view paid more attention to formal aspects; the former view was more critical of the current nosographic identification and classification systems; the latter view reflected the conviction that the IHS classification has always been thoroughly exhaustive since its first 1988 edition [4].

In the next 20 years, the authors who originally embraced either view continued to investigate the issue of CDH in general and of its subtypes in particular; while clinging to their initial beliefs, they were nonetheless willing to pay attention to their counterpart’s opinions and advances.

This intelligent, non-dogmatic approach led to very important, albeit not yet conclusive, achievements.

We will first deal with the milestones that led to the current state of knowledge on CDH in general and chronic migraine in particular. The current official view of the issue will be expounded with all relevant critical points and a proposal will be advanced for changes and adjustments to the classification of the main CDH subchapter: the one about migraine that evolves unfavourably over time.

## Milestones to the current notions of CDH and chronic migraine

Mathew et al. [5] in 1982 were the first to call attention to the possibility that migraine may be transformed into daily headache over time.

Five years later, the same group of researchers [6] reported a case series of 630 CDH patients and attempted to subdivide them into three different groups depending on their headache type: “Type I starts as daily or near-daily headache with no change in the severity and lacks migrainous features; Type II starts as daily or near-daily headaches with occasional more severe headache with some migrainous features; Type III (transformed or evolutive migraine) starts as a clear-cut occasional episodic migraine …. with increasing frequency over the next many years ….. evolving into chronic daily headaches”. As many as 489 of the 630 CDH patients of Mathew et al. (77.7%) belonged to Type III, while only 57 (9.0%) and 84 (13.3%) belonged to Type II and Type I, respectively. These authors in 1987 were not yet talking about chronic tension-type headache (CTTH), nor about new daily persistent headache (NDPH), a form of primary headache first described just 1 year earlier by Vanast [11]. Both were still unknown definitions, even though the two first CDH types could seemingly be identified with these two headache forms. However, back then they already suggested the term “transformed or evolutive migraine” for the third type.

Mathew [12] himself in 1993 definitely chose the name “transformed migraine” (TM), which he included in the first true CDH classification, alongside CTTH and NDPH (Table 1).

**Table 1:** Classifications of chronic daily headache introduced in the mid-1990s

Mathew [12]
1. Transformed migraine
2. Chronic tension-type headache
3. New daily persistent headache
Silberstein et al. [13]
1. Transformed migraine
1.1 With medication overuse
1.2 Without medication overuse
2. Chronic tension-type headache
2.1 With medication overuse
2.2 Without medication overuse
3. New daily persistent headache
3.1 With medication overuse
3.2 Without medication overuse
4. Hemicrania continua
4.1 With medication overuse
4.2 Without medication overuse
Manzoni et al. [14]
1. Evolution of migraine
1.1 Migraine with interparoxysmal headache
1.1.1 Migraine with interparoxysmal headache fulfilling the criteria for chronic tension-type headache
1.1.2 Migraine with interparoxysmal headache not fulfilling the criteria for chronic tension-type headache
1.2 Chronic migraine
2. Chronic tension-type headache

The following year, Silberstein, together with Lipton, Solomon and Mathew again [13], proposed that hemicrania continua (HC) should be added to these three forms and that each of the four forms thus identified should be distinguished depending on the presence or absence of medication overuse (Table 1). They also set precise diagnostic criteria for each form. TM criteria are reported in Table 2.

**Table 2:** Diagnostic criteria for transformed migraine by Silberstein et al. [13]

A. Daily or almost daily (>15 days/month) head pain for >1 month
B. Average headache duration of >4 h day (if untreated)
C. At least 1 of the following:
1. History of episodic migraine meeting any HIS criteria 1.1–1.6
2. History of increasing headache frequency with decreasing severity of migrainous features over at least 3 months
3. Headache at some time meets HIS migraine criteria 1.1–1.6 other than duration
D. Does not meet criteria for new daily persistent headache or hemicrania continua
E. At least 1 of the following:
1. There is no suggestion of one of the disorders listed in groups 5–11
2. Such a disorder is suggested, but it is ruled out by appropriate investigations
3. Such a disorder is present, but first migraine attacks do not occur in close temporal relation to the disorder

In 1995, following the description of a broad case series of CDH patients seen at the Parma and Pavia headache centres in Italy, Manzoni et al. [14] first introduced the name “chronic migraine” (CM), which they included, alongside migraine with interparoxysmal headache (MIH), within the migraine forms that evolve unfavourably over time until they lose the typical symptom-free interval between an attack and the next (Table 1). According to the Italian authors, CM and MIH differentiate from each other for the type of headache that sets in the originally free intervals between attacks: while retaining the clinical features of migraine in CM, in MIH this interval headache loses its similarities to migraine: in some cases it has the same features of tension-type headache, but in other cases it has undefined features and this prevents it from being definitely included in the migraine or the tension-type headache group.

The proposals introduced in the mid-1990s by the US authors [13] and the Italian authors [14] differ from each other in several respects. First, they use different approaches to identify CDH entities: while Silberstein et al. [13] adopt a more detailed and exhaustive classification that includes also NDPH and HC, Manzoni et al. [14] prefer a classification that takes into account only the forms most frequently encountered in clinical practice. Secondly, and more importantly, the differences between them concern: (a) the terminology used to describe migraine forms that evolve unfavourably over time (TM for Silberstein and CM for Manzoni); (b) the temporal requirements for these headache forms (presence of headache at least 15 days per month for at least 1 month for Silberstein and at least 6 days per week for at least 1 year for Manzoni); and (c) Manzoni et al.’s attempt to identify possible clinical subtypes in the group of migraine forms evolving unfavourably (CM and MIH with interval headache with or without tension-type headache features).

If we browse the scientific CDH literature produced from the mid-1990s to as late as 2006, we can see how predominant Silberstein et al.’s systematization became. Almost all studies conducted over this long period of time and aimed at defining the epidemiological, pathogenetic and therapeutic aspects of CDH basically followed Silberstein et al.’s classification [13], both in terms of headache forms included in the classification, and in terms of their respective diagnostic criteria and the terminology proposed to give an official name to each form. In this connection, it is worth noting that there was a general and widespread acceptance of the TM name.

On the other hand, we are sorry to say that the 2004 edition of the International Classification of Headache Disorders classification (ICHD-2) [15] only partially—and not always appropriately—integrated those considerations in its final changes over the 1988 first edition of the IHS classification [4].

In the first place, the ICHD-2 [15] editors did not deem it advisable to devote a separate chapter to CDH within their classification—which, all things considered, is actually a decision we can agree on.

Secondly, of the four different CDH forms identified by Silberstein et al. [13] (Table 1), only one, CTTH, was already included in the 1988 first edition of the IHS classification [4] and then again in the 2004 ICHD-2 classification [15]. Two other forms, HC and NDPH, did not appear in the IHS classification [4] but were included in the ICHD-2 classification [15], where they were coded to Group 4 “Other primary headaches”. The last of Silberstein et al.’s [13] CDH forms, TM, was not recognized as such in the ICHD-2 [15], which however included for the first time CM and its diagnostic criteria, coded to 1.5.1 as a complication of migraine (Table 3). Thus, with respect to the most important and certainly most frequent of all CDH forms, i.e. migraine evolving unfavourably over time, the ICHD-2 [15] eventually retained the CM name, originally proposed by Manzoni et al. [14], but with diagnostic criteria (>15 days per month for at least 3 months) that are very different from those suggested by the Italian authors (at least 6 day per week for at least 1 year). In addition, the ICHD-2 [15] ignored the MIH proposed by the same authors [14]. The result is that only for some of the patients affected by this clinical entity—precisely those with an interval headache resembling tension-type headache—can the ICHD-2 [15] provide a diagnosis, but even so it would be a dual diagnosis, of migraine without aura and of tension-type headache. MIH patients with an interval headache not resembling either migraine or tension-type headache remain unclassified.

**Table 3:** Diagnostic criteria for chronic migraine by the ICHD-2 (2004) and by the ICHD-2R (2006)

ICHD-2 (2004)
A. Headache fulfilling criteria C and D for migraine without aura on ≥15 days per month for >3 months
B. Not attributed to another disorder
ICHD-2R (2006)
A. Headache (tension-type and/or migraine) on ≥15 days per month for ≥3 months
B. Occurring in a patient who has had at least five attacks fulfilling criteria for 1.1 Migraine without aura
C. On ≥8 days per month for ≥3 months headache has fulfilled C.1 and/or C.2 below; that is, has fulfilled criteria for pain and associated symptoms of migraine without aura:
1. Has at least two of a–d:
a. Unilateral location
b. Pulsating quality
c. Moderate or severe pain intensity
d. Aggravation by or causing avoidance of routine physical activity (e.g., walking or climbing stairs)
And at least one of a or b below:
a. Nausea and/or vomiting
b. Photophobia and phonophobia
2. Treated and relieved by triptan(s) or ergot before the expected development of C.1 above
D. No medication overuse and not attributed to another causative disorder

With respect to Silberstein et al.’s classification [13], the ICHD-2 classification [15] neither recognizes the TM definition nor agrees with its diagnostic criteria (Tables 2, 3). Additionally, the ICHD-2 [15] does not envisage, either for CM or even for CTTH, HC and NDPH, a differentiation based on the presence or absence of medication overuse, because it prefers to retain Medication-overuse headache as an autonomous clinical entity (coded to 8.2 of the 2004 classification), much as the 1988 IHS classification [4] had already done.

As defined by the diagnostic criteria of the ICHD-2 classification [15], CM seems: (a) to resemble more a high-frequency migraine than a migraine evolving over time to a daily or near-daily form and eventually losing some, and in certain cases, many of its typical migraine features, such as unilateral and/or throbbing pain and/or nausea and vomiting as accompanying symptoms; (b) to be scarcely relevant to actual clinical practice, because a patient with more than 15 days of headache per month is highly unlikely to use symptomatic drugs for less than 10–15 days per month.

Since its inclusion in the ICHD-2 classification [15], then, CM has always appeared as an ambiguous clinical entity and one that would not be of much use either for clinical practice or research.

Bigal et al. [16] in the US tried to re-classify 638 CDH patients seen over a period of 20 years at the New England Medical Center for Headache in Stamford, Connecticut. By strictly applying the diagnostic criteria of the ICHD-2 [15], they managed to establish a diagnosis of CM only in nine cases. In contrast, using the diagnostic criteria proposed by Silberstein et al. [13], the number of patients that in the same case series could be classified as suffering from TM without medication overuse would be as high as 158. Therefore, the authors concluded that CM diagnosis, as defined and formally applied in the ICHD-2 [15], does not offer any considerable benefit to the CDH diagnostic issue.

Another major shortcoming of the ICHD-2 classification [15] is that, as was clearly demonstrated in the same analysis by Bigal et al. [16], most CDH patients receive multiple diagnoses, in several cases as many as four or five, including some that are only probable. Since the very introduction of the ICHD-2 [15] in 2004, then, it has been clear that the diagnostic criteria of CM needed to be changed.

## Current official stance about CM: merits and critical issues

Even the Committee that worked them out was aware of this problem and new criteria were formulated as a result (ICHD-2R) [17]. Today, these criteria (Table 3) are considered standard reference and, according to Olesen, all of them could be integrated in the future ICHD-3 classification [18, 19].

This is undoubtedly a significant step forward. The new criteria [17] are much more relevant to actual clinical practice than the previous ones of the ICHD-2 classification [15] and may provide a good starting point to get to a better understanding of this complex and all-important chapter of headaches [20–22].

Nevertheless, a few questions remain to be solved and these basically concern two aspects that the headache clinician knows well and the headache researcher cannot underestimate.

The first major question is that a severity gradient exists in CM, as defined in the ICHD-2R classification [17] diagnostic criteria, and it is so wide-ranging as to carry the risk of including exceedingly different cases under the single group of CM [23]. Patients who for 3 months have had 8 days of migraine and 7 days of tension-type headache per month have almost nothing to do with patients who have suffered from migraine-like headache every day for years. The former have a good chance of improving, if adequately treated; the latter generally do not respond well to standard treatments and require a special, customized therapeutic approach. In other words, the minimum time limits set in the ICHD-2R [17] for diagnosis of CM (15 days per month for 3 months) appear to be too short to allow inclusion of CM itself among complications of migraine. These might actually be cases of migraine with a transiently high frequency or of a transient association of medium-frequency migraine and medium-frequency tension-type headache, as can often be seen in the natural history of migraine. However, none of these cases would represent a true complication of migraine. Creating a single loose-net subchapter of headaches, as would be the case with CM according to the ICHD-2R [17], will likely be of no use either to the clinician or to the researcher or even the drug trial investigator. Indeed, it could ultimately jeopardize the key goal of all those who are concerned with the issue, i.e. providing both basic and clinical research with a reliable instrument that can be effectively used to collect homogeneous and well-defined case series. If the tool we are using does not allow an adequate selection of the case series under study, the results obtained—whether from epidemiological surveys, pathogenetic investigations or drug trials—will be misleading and the effort made to achieve them useless [24–29].

The second major question is the problem of symptomatic medication overuse, which affects most patients with CM [30–35]. As the IHS classification [4] before it, the ICHD-2 classification [15] also includes Medication-overuse headache in Group 8 (“Headache attributed to a substance or its withdrawal”). The ICHD-2 [15] diagnostic criteria were revised twice in the last few years and those that are currently recognized as valid (ICHD-2R) (Table 4) for diagnosis of Medication-overuse headache no longer require that headache resolve or revert to its previous patterns within 2 months after discontinuation of the overused medication [17]. Thus, according to the ICHD-2R classification [17], a patient with a form of migraine that has worsened over the years or has become complicated evolving into daily or near daily, with daily or near-daily use of symptomatic drugs, should have a dual diagnosis: Medication-overuse headache and Probable CM. Apart from the drawbacks of multiple diagnoses, it is difficult to see a patient with CM who will not use drugs to block, or at least relieve pain, and if the headache recurs at least 15 days per month—as must happen for the diagnosis of CM to be established—this patient will likely report medication overuse as a consequence, if not necessarily as a cause. Instead of a dual diagnosis of Medication-overuse headache and Probable CM, would not a single diagnosis of CM with medication overuse be preferable in such a patient until he/she is freed from the overused drugs?

**Table 4:** Diagnostic criteria for Medication-overuse headache by the ICHD-2R (2006)

A. Headache present on ≥15 days/month
B. Regular overuse for >3 months of one or more acute/symptomatic treatment drugs as defined under subforms
1. Ergotamine, triptans, opioids, or combination analgesic medications on ≥10 days/month on a regular basis for >3 months
2. Simple analgesics or any combination of ergotamine, triptans, analgesic opioids on ≥15 days/month on a regular basis for >3 months without overuse of any single class alone
C. Headache has developed or markedly worsened during medication overuse

The uncertainties about Medication-overuse headache are also related to the lack of specific scientific data on the role that the individual symptomatic drugs taken by migraineurs might play on the underlying course of the disease, if overused.

As things stand now, we cannot but agree with what was recently stated by Bigal et al.: “The ultimate question that needs to be discussed by the scientific community is not how to better classify migraine overuse headache, but if migraine overuse headache should exist as a single entity or is more appropriately viewed as a risk factor” [36].

## A new proposal for classification of CM

The next edition of the ICHD classification (ICHD-3), due out by the end of 2012, will certainly introduce some changes to CM as defined in the ICHD-2 classification [15] (Table 3).

It may be that for CM the ICHD-3 will merely replace the much criticized diagnostic criteria of the ICHD-2 [15] with the more acceptable ones proposed by the ICHD-2R [17] (Table 3). This would certainly be a significant step forward over the ICHD-2 [15], but a few questions remain unsolved.

The term “chronic migraine” seems ambiguous and inaccurate. Olesen himself [37] criticized the use of the adjective “chronic”, which, as was reported in a recent article by Seshia et al. [38], is given three different meanings in the ICHD-2 [15]. The term “transformed migraine” is to be preferred, because it is less ambiguous and more indicative of the type of patients we are referring to [12, 13, 39–41].

Even though we avoid use of the adjective “chronic”, in order to establish a diagnosis of TM we nonetheless have to set a minimum period of time for the duration of this daily or near-daily headache pattern. The 3 month period generally taken as reference until now seems too short and carries the inherent risk of considering TM as a form of migraine that merely undergoes an entirely transient worsening. A 1 year period seems more appropriate [42, 43].

The same applies to the other temporal parameter, which must define and therefore better specify the vague expression “daily or near-daily” originally used by Mathew et al. [5, 6]. Quantifying this daily or near-daily parameter as ≥15 days appears an oversimplification. In order to avoid too loose a categorization, a more accurate statement would be ≥20 days/month, adding also that there are never >5 headache-free consecutive days [44].

Then, there is the big question of Medication-overuse headache. From the IHS classification [4] to the ICHD-2 [15] and ICHD-2R [17] classifications, the question has become increasingly confused [33, 39–42]. The addition of triptans, the addition of overuse from other symptomatic drugs, the addition of the limits of 10 days/month for overuse from certain drugs and 15 days/month for overuse from other drugs, the addition and then the removal of the criterion that required improvement within 2 months (!) from withdrawal of medication, the addition and then the removal of a description of certain headache features for some types of overuse… All these tentative changes clearly indicate that we are still a long way from knowing this topic well, because literature reports are still much too scarce. As is currently categorized in the ICHD-2 [15] or proposed in the ICHD-2R [17], Medication-overuse headache today is a largely arbitrary entity and its very existence appears questionable for certain subtypes. The only sure data we have are those about ergotamine, caffeine and combined medications containing barbiturates or codeine and, partly, triptans. Waiting for future specific studies to yield more reliable results than are available today and help clarify whether Medication-overuse headache should be considered less an autonomous entity than a complication of migraine [45], at the current state of knowledge perhaps we should better take a more cautious approach to this chapter.

A basic reason that could explain why, in spite of the efforts made by so many authors for so many years, we have not yet come to share a common systematization for the classification of migraine that evolves unfavourably, is that international classifications—the IHS of 1988 [4] and the ICHD-2 of 2004 [15]—are typically classifications of headache attacks, providing a “snapshot” of a headache attack at a given moment. For primary headaches at least, they are not and do not purport to be classifications of disorders or of patients. This approach certainly has merits and advantages and such classifications have become indispensable tools for epidemiological, pathophysiological and therapeutic research. However, they are not suitable for headache forms such as CM or TM, in which what matters most is the patient’s history, and not a snapshot of his/her headache, which can only be blurred and confused.

Hopefully, in the future we will be able and willing to engage in the preparation of a classification of headache syndromes that may combine all current international headache classifications. This is an ambitious and difficult project, which will require several years to complete, but could allow an adequate and correct categorization of patients with CDH.

Waiting for this goal to be achieved, based on the current state of knowledge and on what was previously discussed and expounded in this paper, we think it reasonable to formulate a simple and practical proposal, which can be broken down as follows:

- differentiation of migraine without aura based on frequency of attacks, with the addition of a third-digit level (Table 5);

**Table 5:** Proposed revision of the ICHD-2 for migraine

1.1 Migraine without aura
1.1.1 Infrequent migraine
1.1.2 Frequent migraine
1.1.3 Chronic migraine
1.1.3.1 With medication overuse
1.1.3.2 Without medication overuse
1.5 Complications of migraine
1.5.1 Transformed migraine
1.5.1.1 With medication overuse
1.5.1.2 Without medication overuse
- shifting of CM from its current coding position in the ICHD-2 and the ICHD-2R among migraine complications to a different coding position as a high-frequency subtype of migraine without aura; CM, coded to 1.1.3 would then represent an evolution stage in the natural chronicization course of migraine;
- introduction of precise temporal parameters among the diagnostic criteria for the three migraine without aura subtypes (infrequent, frequent and chronic) (Table 6);

**Table 6:** Proposed diagnostic criteria for the three migraine without aura subtypes, and for transformed migraine

1.1.1 Infrequent migraine
A. Headache fulfilling criteria C and D for 1.1 Migraine without aura on ≤3 days/month for ≥3 months
B. Not attributed to another disorder
1.1.2 Frequent migraine
A. Headache fulfilling criteria C and D for 1.1 Migraine without aura on >3 but <10 days/month for ≥3 months
B. Not attributed to another disorder
1.1.3 Chronic migraine
A. Headache fulfilling criteria C and D for 1.1 Migraine without aura on ≥10 but ≤20 days/month for ≥3 months
B. Not attributed to another disorder
1.5.1 Transformed migraine
A. Headache (tension-type and/or migraine) on >20 days/month for ≥1 year and never with more than 5 headache-free consecutive days
B. Occurring in a patient who has had at least five attacks fulfilling criteria for 1.1 Migraine without aura
C. On ≥10 days per month for ≥1 year headache has fulfilled criteria for pain and associated symptoms of migraine without aura or patient has been successfully treated with an ergot or triptan
D. Not attributed to another disorder
- inclusion of TM among the complications of migraine: TM should be coded to 1.5.1 replacing CM (Table 5) and its diagnostic criteria should be different from those listed in the ICHD-2R [17] only in as far as frequency and duration are concerned (>20 days/month for ≥1 year and never with >5 headache-free consecutive days) (Table 6);
- differentiation of TM at the fourth-digit level depending on the presence or absence of symptomatic medication overuse (Table 5) (i.e. use for >20 days/month) regardless of whether overuse played any role in the worsening of the headache;
- shifting of Medication-overuse headache to the Appendix with alternative diagnostic criteria to be defined.
